# Use of Supplemented Medical-Grade Honey to Treat Traumatic Skin Injuries in Geriatric Patients in a Home-Care Setting

**DOI:** 10.7759/cureus.80189

**Published:** 2025-03-07

**Authors:** Georgios E Papanikolaou, Pieter Kegels, Georgios Gousios, Filip Kegels, Linsey Peters, Niels Cremers

**Affiliations:** 1 Plastic Surgery, Plastic Surgery Private Practice, Ioannina, GRC; 2 Wound Care, Thuisverpleging Kegels, Sint-Gilles-Waas, BEL; 3 Wound Care, Pharmalife, Ioannina, GRC; 4 Gynaecology and Obstetrics, Maastricht University Medical Centre, Maastricht, NLD; 5 Wound Care, Triticum Exploitatie BV, Maastricht, NLD

**Keywords:** antimicrobial effect, geriatric population, medical-grade honey, skin injury, traumatic wound, wound healing

## Abstract

Background

Traumatic skin injuries (TSI) are more common to geriatric population due to reduced skin elasticity and increased gait instability. This is also associated with an altered wound-healing mechanism thus requires a cost-efficient and effective treatment. Therefore, the aim of the present study is to highlight the efficacy of medical-grade honey (MGH) supplemented with vitamins C and E for the conservative treatment of TSI in a home-care setting.

Methodology

The present multicenter retrospective case series study included 10 geriatric patients (four men and six women) who sustained TSI of various etiologies. The median age of the patients was 82.5 years (min-max: 65-90 years). Cardiovascular disease was the most frequent concomitant illness (50% of the patients). Previous treatments with povidone-iodine, alginate gel, or simple gauze for 5.5 days (median, min-max: 0-21 days) were ineffective. Treatment with a variety of supplemented MGH products, including ointment, wound gel, gauze, and foam, was commenced upon the initial patients’ examination.

Results

By using MGH products, we achieved effective reduction of the inflammation, removal of the necrotic tissue, and formation of healthy granulation and epithelial tissue. MGH eliminated clinical signs of infection after 7.5 days (median, min-max: 5-35 days). Wounds were completely healed after 21 days (median, min-max: 14-56 days), without evidence of recurrence or complications, and with good functional and aesthetic outcomes.

Conclusions

Supplemented MGH-based products present high clinical efficacy for the treatment of TSI in older adults in a home-care setting, while demonstrating a safe and easy-to-use profile. Therefore, they can be proposed as an alternative or complementary therapeutic approach to conventional TSI therapies.

## Introduction

Traumatic skin injuries (TSI) are a wide variety of acute, non-surgical wounds, including lacerations, skin tears, burns, abrasions, and bite wounds [1,2]. Although TSI are classified as acute wounds, patients above the age of 65 years have an increased risk of developing chronic wounds due to comorbidities and aged skin [3-6]. Compared to acute wounds, chronic wounds pose a significant economic burden due to their prolonged existence and requirement for more resources [7]. Additionally, geriatric patients are more susceptible to developing TSI due to reduced skin elasticity and increased gait instability [5,6,8]. Therefore, TSI in this patient group are a massive burden on the healthcare system, particularly for home care or community care. Chronic wounds, if left untreated, can contribute to an increased risk of complications, creating a potentially vicious cycle [9].

Current TSI treatment strategies generally involve cleansing the wound, directly suturing the wound if possible, and preventing infection using povidone-iodine, silver-based dressings, or antibiotics [10,11]. However, these methods have significant drawbacks such as the risk of developing antimicrobial resistance and cytotoxicity [12,13]. Besides the antimicrobial effects that lead to improved wound healing, none of these treatment options directly stimulate the wound-healing processes [13,14]. Given the high prevalence of comorbidities in older adults, such as diabetes and vascular insufficiency, that can delay wound healing, TSI should ideally receive a treatment that speeds recovery while also providing antimicrobial effects. Additionally, wound care products should be easy to use and cost-effective.

Honey has been used throughout history for various medical conditions, including wounds. Honey used nowadays in wound care is produced and processed under rigorous conditions, to ensure safe and effective use in medical settings [15]. This type of honey is referred to as medical-grade honey (MGH) [15].

MGH is known for its antimicrobial and wound-healing properties. Its antimicrobial effects are based on its acidic pH, its osmotic action, the formation of hydrogen peroxide, and the action of antimicrobial agents [16-18]. Just as its antimicrobial mechanisms, MGH stimulates wound healing in a multifaceted manner. Its osmotic effects stimulate autolytic debridement and create a moist wound environment. Moreover, MGH stimulates angiogenesis, provides nutrition to the wound, and has anti-inflammatory and antioxidant properties [19,20]. All the aforementioned aspects together result in a faster wound-healing trajectory. The supplementation of vitamins and carrier compounds enhances the antimicrobial and pro-healing effects of MGH, providing practical benefits [19,21-23].

Supplemented MGH has been used successfully to treat wounds in geriatric patients before [24-31]. Although MGH has previously been used in older adults, data on its use for treating TSI in a home care setting is limited. Therefore, through this multicenter observational case series, we aimed to highlight the effectiveness of supplemented MGH products for treating TSI in older adults in a home-care setting.

A version of this manuscript was previously presented as an e-poster at the 34th European Wound Management Association (EWMA) Conference on May 3, 2024, in London, United Kingdom.

## Materials and methods

Study design

The study was conducted in accordance with the principles presented in the Declaration of Helsinki and relevant ethical national guidelines, and the protocol was designated and approved by all the involved parties (protocol code: #2024-L-M-03, approval date: March 27, 2024, L-Mesitran for treating traumatic wounds in the elderly; approved by the research department of Triticum Exploitatie BV, Maastricht). The primary purpose of the present study was to assess the healing and antimicrobial effects of MGH products in the elderly population. Moreover, we aimed to demonstrate the safety, ease of use, and cost-effectiveness of MGH products. All the above objectives were explained in detail to the patients and their relatives, for which they gave their informed consent.

In the present multicenter (Georgios Papanikolaou's (GP) Plastic Surgery Private Practice, Ioannina, Greece, and Thuisverpleging Kegels Wound Care, Sint-Gilles-Waas, Belgium) retrospective case series study, we included 10 patients (four men and six women) 65 years or older with wounds of traumatic origin. Additional inclusion criteria were the presence of wounds for less than four weeks, and failure of previous treatment methods, such as the local use of different dressings and antiseptic preparations. Patients or their relatives who did not consent to participate, who had chronic wounds (present for four weeks or longer), or who were allergic to MGH were not included in the study.

Subsequently, all patients received MGH as monotherapy, except for one who also received antibiotics due to the risk of systemic infection after a dog bite. The TSI were treated by a wound care professional at the patients’ home using a variety of MGH products based on the clinical appearance of the wounds. Wounds with necrotic tissue were treated with L-Mesitran Ointment (L-MO) because this product has the strongest debridement activity. All other wounds were treated using L-Mesitran Soft (L-MS) wound gel, whether there was clinical evidence of infection or not. Moreover, L-MS was used to ensure contact with the wound bed when combined with L-Mesitran Tulle (L-MT). L-MT was used to enhance the activity of the L-MS and to avoid adherence of the secondary dressing to the wound bed. L-Mesitran Foam (L-MF) was used for wounds with moderate and heavy amount of exudate and for its cushioning effect to protect against additional mechanical damage.

The wound-healing progress was monitored through clinical evaluation of tissue viability, infection resolution, wound size reduction, and appearance of healthy granulation and epithelial tissue, until full healing via photographic documentation. Overall patient conditions during treatment, including complications due to treatment and pain, were monitored by the wound care professional. 

The data was collected and analyzed using the Microsoft Excel spreadsheet software, version Office 16.0 for Windows (Microsoft Corporation, Redmont, WA), and incorporated formulas were used accordingly to determine the medians and standard deviations.

Medical-grade honey products

Triticum Exploitatie BV (Maastricht, The Netherlands) produces a variety of MGH-based products tailored for diverse skin wounds, including wounds of traumatic origin. One of their key products is L-MS, an antimicrobial wound gel containing 40% MGH, vitamins C and E, lanolin, propylene glycol, and PEG4000 [32]. Another product from their range is L-MO, an ointment consisting of 48% MGH, lanolin, vitamins C and E, zinc oxide, Aloe barbadensis, and essential oils. Both L-MS and L-MO create a moist wound-healing environment, stimulate debridement of the necrotic material, and inhibit microbial colonization, thereby promoting the overall wound-healing process [20,29,30,32]. The product range also includes dressings, including L-MT, a non-adhering antibacterial dressing impregnated with L-MS gel. L-MT is used to treat wounds of various grades of depth and inflammation and is convenient to apply. The L-MS layer precludes the integration of the dressing into the neo-epithelial and granulation tissue on the wound bed. Another dressing is the L-MF, which is a non-adherent polyurethane foam dressing impregnated with L-MS. The L-MF provides wound healing and antimicrobial effects of honey while managing moderate to high levels of exudate.

## Results

General population data

The demographic data and wound-healing parameters of the population presented in this case series are summarized in Table 1. The median age of the patient population was 82.5 years (min-max: 65-90 years) with a standard deviation (SD) of nine years. Furthermore, cardiovascular disease such as heart disease or arterial hypertension were present in five out of 10 patients. Most wounds were in the lower extremities as eight out of 10 patients presented with wounds on either the legs or feet. Moreover, wounds were present for a median of 5.5 days (min-max: 0-21 days, and SD: 7.1 days), and five out of 10 wounds received treatment with povidone-iodine before the MGH therapy. A healthy wound bed appeared after a median of 7.5 days (min-max: 5-35 days, and SD: 11.4 days), and full healing was achieved after a median of 21 days (min-max: 14-56 days; SD: 17.7 days). All wounds healed uneventfully without any complication and evidence of recurrence during the patients’ period of follow-up.

**Table 1:** Patients’ data and wound characteristics overview of the presented cases

Patient (sex)	Age (years)	Wound etiology	Comorbidities	Location	Wound age (days from wound appearance to MGH-therapy start)	Previous treatment	Time for appearance of healthy tissue (days)	Wound healing time (days)
Case 1 (F)	78	Accidental fall - laceration	Giant cell arteritis with polymyalgia rheumatica, AHT, osteoporosis, vertigo, depression	Anterior surface Left leg	14	Povidone-iodine	28	56
Case 2 (F)	68	Chronic pruritus	PAD, DM type II, dystonia, hypertensive heart disease with congestive heart failure, hyperuricemia	Anterior surface Right leg	21	None	7	21
Case 3 (F)	81	Accidental fall - skin tear with full-thickness skin loss	Dementia in Alzheimer disease, DM type II, vitamin D deficiency	Anterolateral surface Left leg	14	Povidone-iodine	35	56
Case 4 (M)	90	Thermal injury	AHT, heart failure, hypothyroidism, hyperuricemia, anemia, ulcerative colitis	Dorsal surface Right foot	14	Povidone-iodine	28	49
Case 5 (M)	65	Traumatic friction	AHT	Plantar surface Left foot	7	Tincture of iodine	7	14
Case 6 (F)	90	Accidental fall - skin tear with partial thickness skin loss	None	Anterior surface Bilateral legs	4	Povidone-iodine	11	27
Case 7 (M)	90	Accidental fall - skin tear with partial thickness skin loss	None	Dorsal surface Left hand	2	Flaminal hydro	6	15
Case 8 (M)	78	Dog bite	Cancer	Dorsal surface Right hand	0	None	5	15
Case 9 (F)	87	Accidental fall - skin tear with partial thickness skin loss	None	Anterior surface Left leg	3	Simple gauze and povidone-iodine	7	14
Case 10 (F)	84	Accidental fall - laceration	Cardiovascular disease, AHT	Anterior surface Right leg	2	Simple gauze	8	21
Median±SD	82.5±9.0	n.a.	n.a.	n.a.	5.5 ± 7.1	n.a.	7.5 ± 11.4	21 ± 17.7
F: female; M: male; AHT: arterial hypertension; PAD: peripheral artery disease; DM: diabetes mellitus; SD: standard deviation; MGH: medical-grade honey; n.a.= not available								

Case presentation examples

Case 1

**Figure 1:**
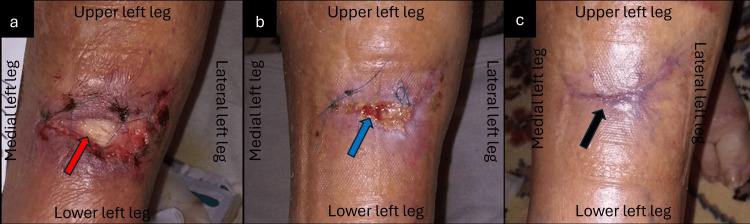
Dehisced surgical wound following primary closure of a full-thickness traumatic leg ulcer (a) Full-thickness wound defect at the anterior area of the left leg, at the beginning of the MGH therapy, with exposure of the tibialis anterior tendon (red arrow); (b) Effective treatment of the local infection, and wound bed coverage with healthy granulation tissue (blue arrow) after four weeks of MGH treatment; (c) The traumatic ulcer healed completely with the appearance of a mature scar (black arrow) after eight weeks of MGH therapy. MGH: medical-grade honey

A 78-year-old female patient presented with a full-thickness traumatic laceration at the anterior area of the left leg after an accidental fall (Figure 1a). The comorbidities included giant cell arteritis with polymyalgia rheumatica, arterial hypertension (AHT), osteoporosis, vertigo, and depression, while she was under antiaggregant (acetylsalicylic acid 100 mg) and glucocorticoid (methylprednisolone 16 mg) therapy. Initially, the injury was sutured and treated with a povidone-iodine solution. After 14 days, the wound presented with dehiscence, exposing the underlying tendon of the tibialis anterior muscle, and concomitant evidence of infection, with moderate quantity of exudate, necrotic tissue, and pain. Treatment was commenced with L-MS wound gel, covered by L-MT and an absorbent pad. The wound care protocol consisted of dressing changes every two days at the patients’ home. With each dressing change, improvements in the wound and reduction of pain were noticeable. After 28 days, the infection was fully resolved, while the wound bed and the tendons were covered by healthy granulation tissue (Figure 1b). Thus, the dressing changes of the wound were extended to every four days, with complete and uneventful healing of the TSI after 56 days (Figure 1c).

Case 3

**Figure 2:**
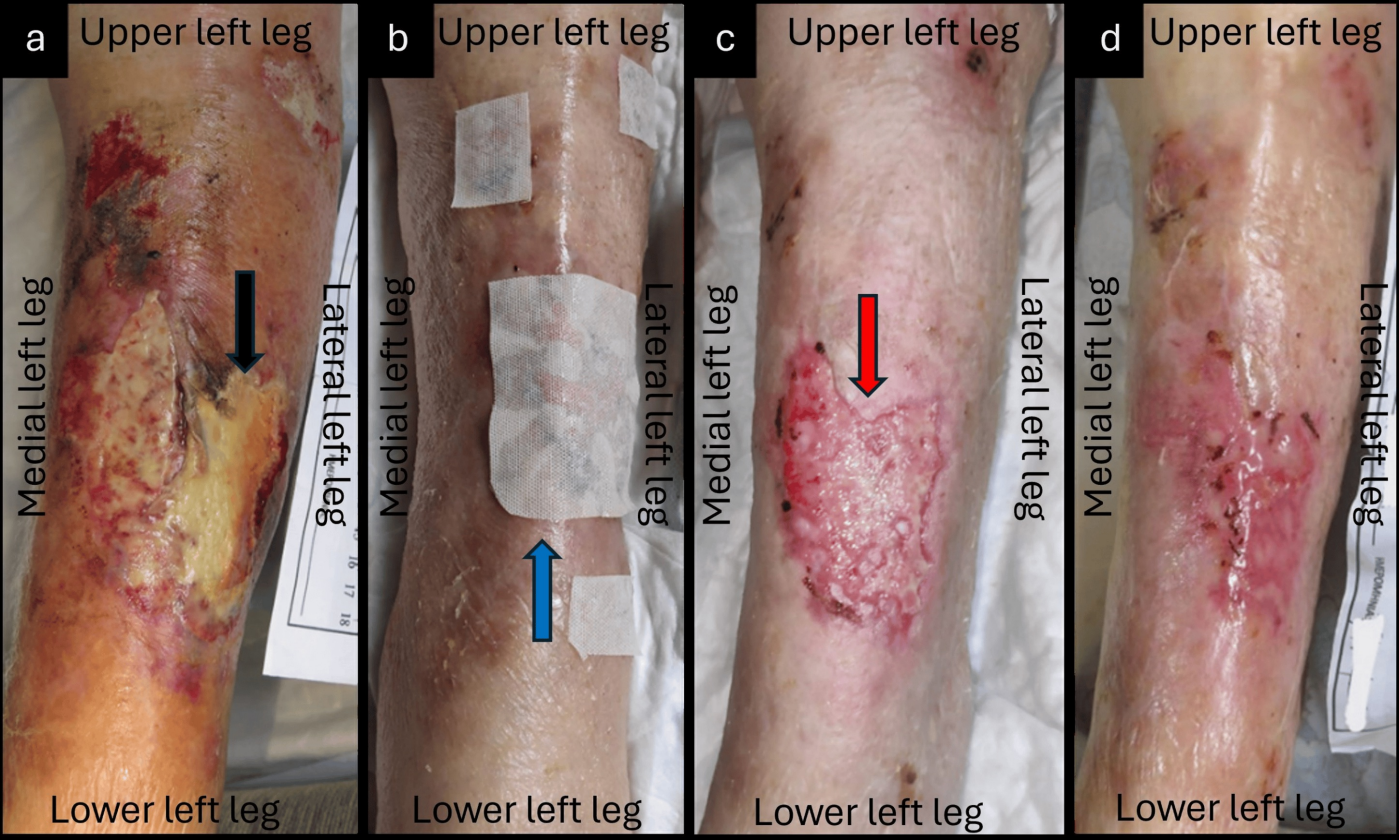
Full-thickness traumatic ulcer at the anterolateral surface of the left leg (a) Clinical findings at the beginning of MGH treatment, with evidence of superficial necrosis and slough (black arrow); (b) Local application of L-Mesitran® Soft wound gel, and L-Mesitran® Tulle (blue arrow); (c) Resolution of the infection, and effective wound healing (red arrow) after five weeks of MGH therapy; (d) The wound was healed uneventfully after eight weeks of MGH therapy. MGH: medical-grade honey

An 81-year-old female patient presented with a skin tear and full-thickness skin loss covering the anterior and lateral surface of her left leg due to an accidental fall at home (Figure 2a). Medical comorbidities included dementia in Alzheimer's disease, type II diabetes mellitus, and vitamin D deficiency. Moreover, the patient suffered from permanent immobility and received antiaggregant (acetylsalicylic acid 100 mg) and glucocorticoid (prednisolone 5 mg) therapy. The TSI was present for about 14 days and treated with povidone-iodinate. On initial observation, in the central area of the wound were present partial thickness necrosis, slough, and a moderate amount of exudate, while erythema, edema, and pain were present at the periphery. Based on the antibacterial and pro-healing effects of MGH, we decided to treat the wound with L-MS wound gel, L-MT, and a secondary absorbent pad. The wound changes were performed every two days in a home-care setting (Figure 2b). During the following 35 days, we noticed the resolution of the local infection, while the wound bed was filled with neo-epithelial and granulation tissue (Figure 2c). After 56 days, the wound showed a significant improvement in the healing process, and complete coverage with healthy and stable scar tissue without further complications (Figure 2d).

Case 4

**Figure 3:**
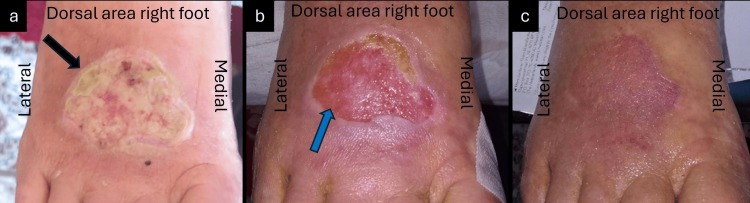
Deep partial-thickness post-burn traumatic ulcer on the dorsal surface of the right foot (a) At the initial presentation, the wound was covered by a superficial slough (black arrow); (b) The ulcer is clear, with appearance of healthy granulation and epithelial tissue (blue arrow) after four weeks of MGH treatment; (c) The wound healed completely after seven weeks of MGH treatment. MGH: medical-grade honey

A 90-year-old male patient presented with a partial-thickness TSI at the dorsal area of his right foot due to a thermal burn (Figure 3a). His medical history included AHT, heart failure, hypothyroidism, hyperuricemia, anemia, and ulcerative colitis. Previous local treatment with povidone iodinate solution for 14 days was ineffective. Upon presentation, the wound presented with slough, moderate levels of exudate, and pain. Based on these clinical findings, we decided to start the local treatment of the wound with L-MS, L-MT, an absorbent foam, and the dressing changes were performed every two days in a home-care setting. Additionally, we advised the patient to keep his lower leg elevated and be ambulatory throughout the treatment. Already after 28 days, the pain had markedly decreased, the slough dissolved, and the wound began to cover with neo-epithelial and granulation tissue (Figure 3b). Therefore, the intervals of wound changes were increased to every four days, and the ulcer showed complete and uneventful healing, with minimal scarring after 49 days of MGH therapy (Figure 3c).

## Discussion

Acute wounds, such as TSI, have a high chance of turning into chronic wounds in older patients. This is because of the higher fragility and slower healing capacity of the older skin and the presence of comorbidities in a geriatric population [3-6]. In the current case series, we presented 10 patients with a median age of 82.5 years (min-max: 65-90 years) who received home care for TSI. Wounds were present for a median of 5.5 days (min.-max.: 0-21 days) and previous treatments, such as povidone-iodine, simple gauze, and enzymatic gel, failed to improve the state of the wound. To prevent the wound from becoming chronic, treatment with MGH was started. All TSI were successfully treated with MGH, leading to fast recovery without further complications and recurrence during the follow-up period.

MGH effectively resolved the infection, as indicated by the appearance of the healthy tissue, after a median of 7.5 days (min.-max.: 5-35 days). MGH was used as a monotherapy, except for one patient who received antibiotics for an increased risk of systemic infection following a dog bite injury. No wound swabs were taken to identify the exact pathogens responsible for the infections. Although the pathogen was unknown, the MGH treatment effectively resolved the signs of clinical infection without using other antimicrobial thereby, emphasizing its wide-spectrum antimicrobial efficacy. The clinical efficacy of MGH against a variety of common wound pathogens has been demonstrated previously in different case series in which the microorganisms were determined [26,30,32]. Besides its antimicrobial effects, its pro-healing activities also were evident in the current case series. This was noticeable by the increased autolytic debridement, reduced inflammation, and the promotion of granulation and re-epithelialization. The wounds were fully healed after 21 days median (min-max: 14-56 days), which is in concordance with other publications [25,26,28-30,32]. A recent meta-analysis demonstrated that honey can effectively reduce wound healing time and rate, incurred pain, hospitalization, and accelerated granulation in diabetic foot ulcers [33]. Similarly, a systematic review reported that honey compared to other products was cost-effective for treating diverse acute and chronic wounds, including burns, venous leg and pressure ulcers, surgical wounds, diabetic foot, and to ensure infection control and reduction of the wound size in malignant wounds [34].

Povidone-iodine is commonly used to treat acute wounds for its wide-spectrum antibacterial effects. It releases free iodine, swiftly infiltrating microorganisms and leading to eventual cell death [35,36]. Although it is beneficial for wound healing in infected wounds, its cytotoxicity and benefits in non-infected wounds are still debatable [37]. Moreover, povidone-iodine can cause temporary skin discoloration preventing the healthcare professional from properly observing changes in the wounds, such as erythema and hyperemia. In case 1, for example, even though the sutured wound was being treated with povidone-iodine, the wound dehisced underlining its lack of pro-healing effects. The bacterial infection was also not resolved at this point, indicating the antimicrobial effects of povidone-iodine don’t last long in time. In a systematic review and meta-analysis by Zhang et al., the wound-healing efficacy of topical honey applications was compared to povidone-iodine-based dressings, and the analysis revealed that honey showed a medium-to-large effect in the reduction of wound-healing time, length of hospitalization, and a visual analogue scale score of pain compared to povidone-iodine [38]. Further highlighting the benefits of honey over povidone-iodine are studies performed on cesarean section wounds [39,40]. One study compared daily honey application to a combination of antibiotics with povidone-iodine (twice a day applied) [39], and the other study compared the combination of honey and povidone-iodine to only povidone-iodine treatment [40], where both clinical trials showed MGH decreased healing time. The effective pro-healing and broad-spectrum antimicrobial activity of MGH, as demonstrated by the aforementioned studies and meta-analysis, supports a more cost-effective approach. The wound-healing process can be completed in its due time, making use of fewer products and fewer visits from healthcare professionals.

The most common comorbidities were arterial hypertension and cardiovascular disease, which were present in half of the patients. Patients with cardiovascular disease or arterial hypertension often have a disturbed or limited flow of blood carrying oxygen and nutrients to the wound site, resulting in slower wound healing [41-44]. Additionally, limb blood flow is reduced with age [45]. Similarly, diabetic neuropathy and peripheral arterial disease can cause the development of lower limb ulcers with the increased risk of delayed wound healing, infection, and eventually limb amputation mainly in older patients [30, 46]. Nonetheless, in our study all TSI were successfully treated with MGH, leading to fast recovery without further complications. Most importantly, the patients treated in a home-care setting and quickly recovered full mobility in their affected limbs, thereby improving their quality of life. Moreover, the products were deemed pain-free and did not cause any trauma upon removal. Products that adhere to or get incorporated into the healing tissue may impede wound repair [47]. Additionally, skin tears also require delicate treatment with a non-adherent wound care product, since viable skin flaps are sensitive to further damage. Overall, all MGH products used in this case series stimulated wound healing while allowing gentle and painless removal from the wound, given their non-adherent properties [32,48].

Although this case series includes practical examples of the application of MGH and presents preliminary results, some considerations should be made. Ideally, swab tests should have been taken to identify the exact pathogen in the wounds and to later confirm the absence of any microbes. However, due to financial reasons and the acute nature of the wound, the swab tests were not performed. Moreover, the diagnosis of infection was based on the clinical presence of local signs of infection, such as exudate, necrosis, malodor, and erythema [29]. Nonetheless, MGH successfully resolved any signs of infection, except for one patient who received antibiotics for an increased risk of systemic infection following a dog bite injury. Moreover, since there is a lack of comparison, the exact wound-healing effects could not be validated. However, the positive outcome of MGH on wound healing has been demonstrated by several systematic reviews and large, controlled trials [35,36,41]. Additionally, we didn’t use objective measurements to evaluate the healing progress, such as wound size and pain level. Instead of that, the wound-healing process was monitored through clinical evaluation of tissue viability, infection resolution, wound size reduction, appearance of healthy granulation and epithelial tissue, and using photographic documentation. Furthermore, case series are considered low-level evidence but provide practical examples that can serve as a basis for future comparative studies. These comparative studies could, for example, examine the effectiveness of MGH compared to other antimicrobials in TSI, focusing on infection and wound healing. Consequently, larger prospective and comparative studies with longer follow-up period are needed to assess the benefits of using MGH products in daily wound management, and specifically in elderly patients in a home-care setting.

## Conclusions

In our case series, supplemented MGH-based wound care products were used to treat TSI of various etiologies successfully in older adults at home. Treatment led to swift infection control and wound closure without any complications due to treatment. Moreover, MGH was easy to use, cost-effective, and improved the patient’s quality of life. MGH can thus be used safely as an alternative or complementary to the standard methods for the treatment of TSI in older adults.
